# The clinical effects of the Össur Power Knee with phase-based and default control during sitting, standing, and walking

**DOI:** 10.1186/s12984-025-01729-2

**Published:** 2025-09-29

**Authors:** T. Kevin Best, C. Andrew Seelhoff, Jeffrey Wensman, Robert D. Gregg

**Affiliations:** 1 Robotics, University of Michigan, 2505 Hayward Street, Ann Arbor, MI 48109 USA; 2 Mechanical Engineering, University of Michigan, 2505 Hayward Street, Ann Arbor, MI 48109 USA; 3 Orthotics and Prosthetics Center, Michigan Medicine, University of Michigan, 2850 S Industrial Hwy, Ann Arbor, MI 48104 USA

**Keywords:** Robotic Prostheses, Prosthesis Control, Transfemoral Amputees

## Abstract

**Background:**

A lack of evidence of compelling clinical benefits is a key factor limiting the adoption of commercialized powered robotic knee prostheses into mainstream clinical practice. Previous studies have demonstrated mixed results, potentially due to a combination of limitations in prosthetic hardware, control algorithms, and testing methodologies.

**Methods:**

We investigated the clinical effects of a commercialized robotic knee prosthesis (the latest generation Össur Power Knee^TM^) with n=7 above-knee amputee participants. Participants with both higher (K4) and lower mobility (K3) completed a series of experiments including repeated sitting and standing, a stand, walk, sit shuttle test, and fast walking on a treadmill. We tested both standard (ÖSSR) and novel (HKIC) control policies and compared the resulting clinical metrics to those found with the users’ prescribed passive prostheses. Our experiments were physically demanding, which could help elucidate the potential benefits of powered knees.

**Results:**

The clinical effects of the Power Knee varied with mobility level and the control policy used. The phase-based controller often produced stronger walking and sit/stand improvements for the higher mobility group compared to the default controller, though it also presented a steeper learning curve and reduced walk-to-sit transition speed. Conversely, the default control policy was perceived as easier to master but was less assistive to the higher mobility group and produced slower sit/stand cycles. Lower mobility participants experienced improvements in standing speed (HKIC: $36.7\pm 15.1$% faster, $p<0.001$; ÖSSR: $28.8\pm 15.1$% faster, $p=0.001$), inter-limb ground reaction force symmetry (HKIC: $-0.214\pm 0.068$, $p<0.001$; ÖSSR: $-0.199\pm 0.068$, $p<0.001$), and inter-limb peak knee moment symmetry (HKIC: $-0.290\pm 0.126$, $p<0.001$; ÖSSR: $-0.284\pm 0.126$, $p<0.001$) during sit-to-stand tasks relative to their passive prostheses. In contrast, higher mobility participants benefited less in sit/stand but showed improvements while walking including increased toe clearance (HKIC: $25.4\pm 12.2$ mm, $p<0.001$; ÖSSR: $13.4\pm 12.2$ mm, $p=0.033$), greater early stance knee flexion (HKIC: $7.1\pm 2.9^\circ$, $p<0.001$; ÖSSR: $4.5\pm 2.9^\circ$, $p=0.005$), and, for the HKIC policy, a reduced swing-phase peak hip flexion moment (HKIC: $-0.18\pm 0.11$ Nm/kg/(m/s), $p=0.003$). Despite these biomechanical improvements and qualitative reports of reduced effort, neither control policy produced significant benefits in endurance or repeated task performance compared to the passive condition. Sit-to-stand cycle count in the lower mobility group was unchanged (HKIC: $p=0.268$, ÖSSR: $p=0.848$), and it was reduced in the higher mobility group with the ÖSSR condition ($2.0\pm 1.4$ fewer, $p=0.007$). In the shuttle walk test, laps completed by higher mobility users decreased with HKIC ($157.3\pm 46.3$ fewer, $p<0.001$), and no significant differences were found for lower mobility users. No significant changes in fast walking distance or speed were observed across conditions.

**Conclusions:**

The latest generation Power Knee can create clinical improvements in walking and sit/stand behaviors compared to passive (microprocessor) knees, though the effects are sensitive to the user’s mobility level and the Power Knee’s control policy. However, these improvements did not directly translate to improved functional performance or endurance. Some negative effects of the Power Knee were also observed including reduced agility, slower transitions, and thermal limitations, though some of these limitations could potentially be addressed through future control innovations or with more thorough acclimation. The observed benefits motivate future longitudinal studies to investigate the clinical effects of robotic knees compared to passive (microprocessor) knees in real-world settings and to elucidate how they could be best utilized in clinical practice.

*Trial Registration*: The experimental protocol was approved by the University of Michigan Institutional Review Board (HUM00230065) on February 9th, 2024. The trial is registered with the National Institutes of Health under ClinicalTrials.gov ID NCT06138977.

**Supplementary Information:**

The online version contains supplementary material available at 10.1186/s12984-025-01729-2.

## Background

Passive knee prostheses, including advanced microprocessor knees (MPKs), are unable to fully replicate the function of the missing limb because of their inability to inject energy and emulate concentric muscle contraction [1]. In contrast, robotic knee prostheses can use their electric motors to add energy as biological muscles do, theoretically providing a more complete replacement for the functions of an amputated limb. Despite this potential, commercialized robotic knee prostheses have yet to achieve widespread clinical adoption. For such adoption to occur, clinicians and reimbursement agencies (e.g., Medicare/Medicaid) would require clear evidence demonstrating that robotic knees could more effectively meet patients’ minimum medical needs or reduce long-term care costs compared to existing standards of care (MPKs) [2–4]. Currently, the real-world clinical advantages of commercialized robotic knees over existing passive knees remain unclear, including whether any benefits sufficiently outweigh potential drawbacks like increased weight, cost, and complexity.

While prototype robotic knees have demonstrated compelling clinical benefits in research settings, these findings have not yet been consistently reproduced in everyday contexts with commercialized devices. For example, prototype robotic knees have been shown to create more normative biomechanics [5–7], improve user symmetry [5, 8–10], and reduce energy expenditure [11, 12], all of which could improve long-term user health if they persisted in everyday use. However, previous studies with commercialized, clinically available robotic knees have not shown these same benefits. For example, Highsmith et al. presented a case study investigating sitting, standing, and walking on the first generation Össur Power Knee^TM^ [13] and an Ottobock C-Leg^TM^. While they observed some improvement in hip moment symmetry while sitting down, they observed no substantial symmetry improvements at the knee while sitting or at any joint while standing. Wolf et al. similarly investigated the biomechanical differences in sit/stand between the second generation Power Knee and the C-Leg, but observed “few differences between components and no effect on the intact limb” [14]. Most recently, Hafner et al. compared clinical outcomes between mechanical knees, microprocessor knees (Össur Rheo Knee^TM^ II), and powered knees (Össur Power Knee, second generation) in an extensive 14-month randomized crossover trial with 12 participants [15]. Users demonstrated generally worse mobility with the Power Knee relative to the Rheo Knee, and the authors noted “high attrition of subjects" in the Power Knee condition, suggesting that users were unhappy with its performance.

This discrepancy in reported outcomes between prototype and commercialized knees may be explained by limitations in hardware, control, and/or testing methodology. The studies in [13–15] were conducted with early versions of the Power Knee, which were heavier than both modern prototypes (e.g. [6]) and the latest generation Power Knee (PKA01). For example, the Power Knee II used in [15] weighed 3.2 kg, while the newer PKA01 weighs 2.7 kg. The extra prosthesis mass could have affected user balance, agility, comfort, and confidence, limiting the observed clinical benefits. Additionally, commercialized knee prostheses often use conservative, discrete-phase control approaches, such as the seminal finite state machine (FSM) impedance controller [16]. While these controllers are highly robust, they can be challenging to tune for optimal performance and may be less adaptable compared to recent data-driven, continuous-phase control approaches [7, 10, 17, 18]. It is possible that the commercialized prostheses in past studies used controllers that were either too conservative or sub-optimally tuned, resulting in less favorable clinical outcomes. Finally, the standard clinical outcome metrics used in past studies of commercialized robotic knees, such as the *Timed Up and Go (TUG)* test, may not be sufficiently challenging for all users. Higher-intensity tasks that challenge and fatigue users could prompt increased reliance on the robotic knee’s assistance, thereby revealing clinical benefits like fatigue mitigation that may otherwise remain hidden in less strenuous experiments [19].

Towards addressing these factors, our preliminary work [20] investigated the clinical benefits of using the latest generation Össur Power Knee (PKA01) in conjunction with a continuous-phase impedance control policy for walking and sit-stand [7, 10]. This version of the Power Knee is lighter compared to its predecessors studied in [13–15], and the phase-based control policy is designed to be more adaptable and user-synchronized than traditional FSM-based control policies without requiring manual tuning [7, 10]. Using this control policy, participants in this previous work [20] demonstrated increased inter-limb sit/stand symmetry and improved walking kinematics with the robotic knee relative to their prescribed passive prostheses. While promising, this previous work was limited to n=4 high-mobility (K4) amputee participants under relatively low-intensity conditions, making it unclear how these results generalize to lower-mobility users and to different activity intensities. Moreover, we did not test the Power Knee using its default control software, leaving it ambiguous whether the benefits were due to the novel control policy or the Power Knee hardware.

In this study, we further investigated the clinical effects of the latest-generation Össur Power Knee with a broader participant pool performing a series of laboratory experiments of varying intensity. We enrolled n=7 above-knee amputee participants, including both higher-mobility (K4) and lower-mobility (K3 requiring walking aides). Our objective was to assess the ways in which the Power Knee affected users’ ability to perform basic activities of daily living, including sitting down, standing up, and walking, relative to their prescribed (passive) prostheses. To isolate the specific effects of the control policy used, we tested both the default FSM-based control policy [21, 22] and a continuous-phase control policy [7, 10]. We evaluated the participants’ performance using clinically-relevant metrics, with the aim of highlighting potential clinical benefits that robotic knee prostheses with modern control policies may offer.

The remainder of this article is organized as follows. In the Methods section, we provide background information on the Power Knee and overview the two control policies used in the study. Then, we discuss the experimental protocol used to evaluate the prostheses. In the Results and Discussion sections, we present the results of the experiments, and discuss their implications for prosthesis design and clinical practice. Finally, the Conclusions section summarizes our conclusions.

## Methods

### Prostheses and control policies under investigation

#### Power Knee hardware overview

In addition to the participants’ prescribed passive prostheses, this study used the latest generation Power Knee from Össur (model PKA01), which is a commercialized robotic knee prosthesis. The drivetrain of the Power Knee consists of a brushless DC motor, a harmonic drive transmission, and a compliant linkage that connects the transmission output to the knee joint. The torque-angle relationship of the compliant linkage is nonlinear, stiffening under increasing deflection. The knee assembly weighs 2.7 kg (including battery) and the joint has a 120 deg range of motion. The removable lithium ion battery powers the motor and the embedded control electronics. The sensing suite includes a joint position encoder, motor position hall-effect sensors, a shank-mounted inertial measurement unit, and an array of ground reaction force (GRF) hall-effect sensors [23].

**Fig. 1:**
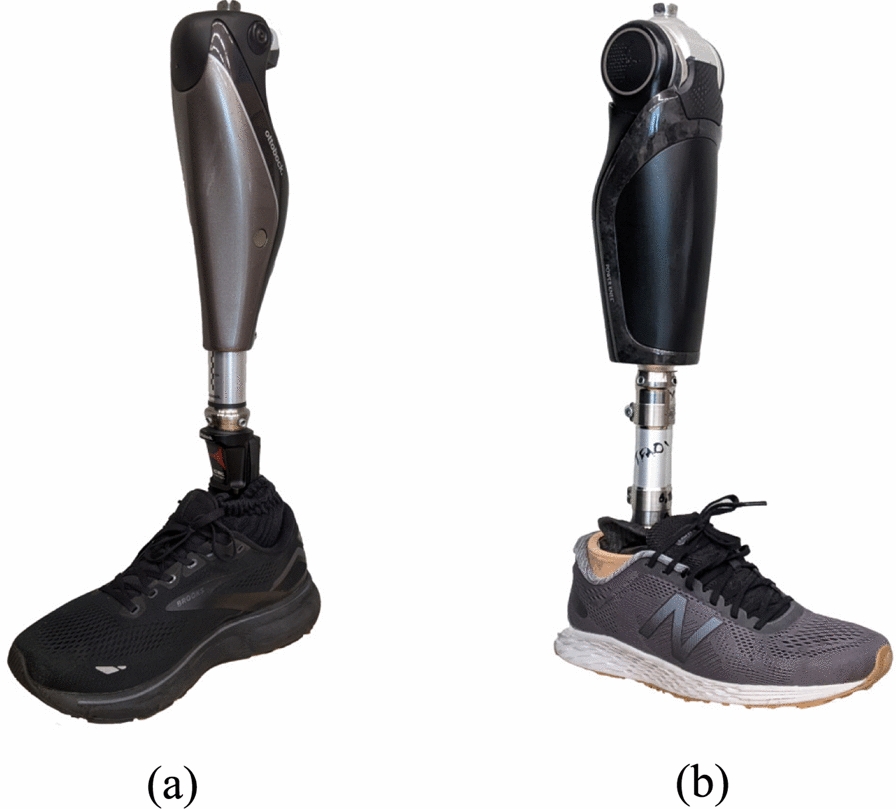
Example photos of the prostheses used in this study. **a** Participant TF01’s prosthesis consisting of an Ottobock Genium MPK with a Ottobock Triton ankle/foot. **b** The Össur Power Knee (PKA01) paired with a Pro-Flex LP foot

Based on the manufacturer’s recommendations, we paired the Power Knee with the Pro-Flex® carbon fiber ankle-foot prosthesis. We used the Low Profile (LP) variety for all participants in order to maximize compatibility with different user heights and limb geometries. The appropriate size and category foot was used for each participant following the sizing guidelines in the Pro-Flex LP Catalog. Averaging across participant configurations, the entire powered prosthetic leg weighed $3.8 \pm 0.1$ kg, including the Power Knee, pylon, foot, shoe, and other hardware components (see Fig. 1).

#### Power Knee control policies

We tested two control policies for the Power Knee in this work: the default control policy from Össur and a custom continuous-phase control policy. The Power Knee’s default controller uses a finite state machine to change a feedback controller’s parameters and produce different locomotion behaviors. Mode transitions (e.g., between walking and sitting) are triggered by user-interpretable cues, and clinicians can adjust various control parameters through a mobile application (see Appendix A.1 for details).

In place of the default control software, we also deployed a simplified version of a multi-activity phase-based controller [19, 24], known as a hybrid kinematic impedance controller (HKIC), for sit/stand and walking (Appendix A.2, Fig. 19). Instead of an FSM, this control policy governs prosthesis joint impedance during stance and joint kinematics during swing using continuous, data-driven models derived from able-bodied demonstration data. The policy modulates the joint behavior based on a continuous phase estimate derived from the user’s residual thigh angle. This implementation of the HKIC for the Power Knee, first presented in [20], integrates sit/stand and walk controllers that were previously developed for prototype robotic knee-ankle prostheses [7, 10]. Key modifications were made to accommodate the Power Knee’s drivetrain dynamics and the passive ankle-foot prosthesis, with complete details provided in Appendix A.2.

### Participant recruitment

We recruited participants with unilateral above-knee amputations from the greater Detroit, MI area to participate in our study. Our inclusion criteria required participants to be between 18 and 70 years old, to regularly use a knee-ankle prosthesis, and to be able to independently ambulate (with or without a mobility aid) for at least 2 months prior to the study. Exclusion criteria included weighing more than 115 kg (due to device limitations), having incompatible limb geometry for the prosthesis, being pregnant, and having other significant locomotion limitations, such as severe arthritis, other major amputations, neuromuscular disorders, or cognitive limitations that could interfere with following directions. Seven participants meeting these criteria were found and enrolled in the study (Table 1). Four of the recruited participants were strong community ambulators (K4) that did not require mobility aids, whereas the remaining three were lower mobility (K3) who often utilized mobility aids such as canes and walkers in everyday life.

**Table 1:** Study cohort information

Participant	Sex	Age (years)	Mass (kg)	Prescribed prosthesis (Knee/Ankle)	Etiology	Amputation type	K-level	Time since amputation (years)
TF01	M	23	79	Genium/Triton	Dysvascular disease	Above knee	4	10
TF02	M	25	84	Rheo/Proflex XC	Congenital	Knee disartic.	4	24
TF03	M	20	75	Quattro/Shockwave	Cancer	Above knee	4	2
TF04	F	42	66	X3/Triton LP	Trauma	Knee disartic.	4	6
TF05	M	69	65	Total Knee/Axtion DP	Infection	Above knee	3	10
TF06	M	55	82	C-Leg 4/Taleo	Medical complication	Above knee	3	5
TF07	F	33	75	Rheo/Variflex LP	Trauma	Above knee	3	2

### Experimental protocol and metrics

**Fig. 2:**
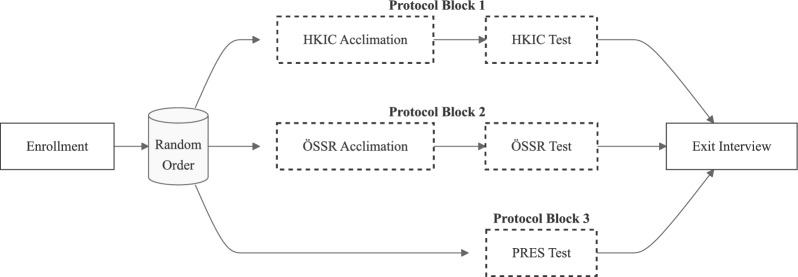
A flowchart depicting the experimental protocol. After enrollment, participants completed three experimental blocks in a randomized order. Each block corresponds to one of the three test conditions (HKIC, ÖSSR, & PRES). The blocks for the HKIC and ÖSSR conditions included acclimation visits to allow participants to learn to sit, stand, and walk with the Power Knee. The activities in each dashed box occurred on separate days to mitigate fatigue effects.

The experimental protocol was approved by the University of Michigan Institutional Review Board (HUM00230065) and each participant provided informed consent. In our study, we evaluated the users’ ability to perform a series of sit/stand and walking experiments under three conditions; participants performed the experiments using either 1) their prescribed prosthesis (PRES), 2) the Power Knee running the phase-based controller (HKIC), or 3) the Power Knee running the default controller from Össur (ÖSSR). Fig. 1 shows photos of the two prostheses used by participant TF01, and the supplemental videos (additional files 1 and 2) show the PRES prostheses of the other participants. The experiment was structured into three protocol blocks, each corresponding to one of the testing conditions (Fig. 2). To prevent procedural bias, the order of these blocks was randomized among participants. Blocks 1 and 2 involved the two Power Knee conditions and Block 3 was dedicated to the PRES condition. Since none of the participants used the Power Knee in their daily lives, we included acclimation visits in Blocks 1 and 2. These visits were designed to teach participants how to sit, stand, and walk with the Power Knee and the specific control policies, allowing them to practice until they felt comfortable.

During the acclimation visits for Blocks 1 and 2, a certified prosthetist fit and aligned the Power Knee prosthesis to each participant. The appropriate Pro-Flex LP foot was used based on the participant’s weight, activity level, and shoe size. Participants received instruction on using the Power Knee with the respective controller and were given ample time to practice sitting, standing, and walking. Training for the ÖSSR condition was conducted according to the steps and best practices provided by the Össur Academy^TM^. Throughout the acclimation session, the research team and prosthetist provided coaching on how to fully utilize the device’s capabilities. The acclimation sessions lasted up to 4 hours.

The three testing visits were identical aside from the prosthesis and controller used (ÖSSR, HKIC, or PRES). After fitting, the participants were allowed to practice sitting, standing, and walking to warm up and re-familiarize themselves with the prosthesis and the specific control policy. Visual feedback during sit-stand was provided to allow the participants to practice symmetric limb loading, similar to [10]. In our experience, this step helps to combat habitual sit/stand asymmetries learned from using passive devices. The visual feedback was removed after the warm up period.

After warm up, the participants completed the main experiment, which comprised three components: a repeated, rapid sit/stand test, an incremental shuttle walk test between two stools, and a fast treadmill walking test. These tests were intended to be challenging and fatiguing, which we believed would encourage the participants to leverage the assistance that a robotic prosthesis could provide. Although these conditions are not exactly similar to how many people would use a robotic prosthesis in everyday life, increasing the intensity of the experiments helps to amplify any fatigue-related signals that vary between the conditions and to observe them during a reasonable-length lab-based experiment. A video file highlighting the experiments is available for download (Additional file 1).

For safety, handrails were provided along the walkway, but their use was discouraged unless required to prevent a fall. The lower mobility participants (TF05-TF07) were permitted to use the handrails during sit/stand if they could not otherwise complete the activity due to poor balance. A ten minute break was enforced between each test, allowing participants to drink water, adjust their prosthetic liners, and mitigate socket sweat before the next experiment.

**Fig. 3:**
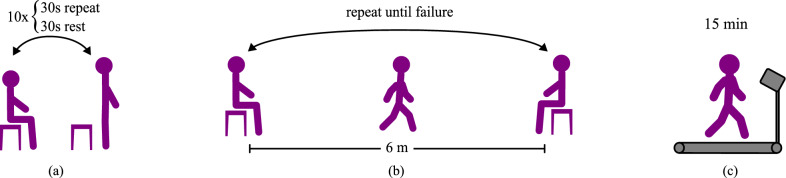
Graphical depictions of the experiments. **a** Participants performed repeated sit/stand cycles as fast as possible for 30 s at a time with 30 s rest intervals for 10 rounds. **b** Participants performed an incremental shuttle walk test involving repeated walking between two stools approximately 6 m apart (illustration modified from [20]). Walking laps between the stools continued until the participants could no longer maintain the cued pace, which slowly increased throughout the experiment. **c** Participants walked on a treadmill at a self-selected fast pace for 15 min

#### Repeated sit/stand test

To assess the users’ ability to complete sitting and standing motions under each condition, we conducted a first test consisting of repeated sit-stand-sit cycles from a 45 cm stool. Participants were asked to complete as many cycles as possible in 30 s while maintaining good form (*i.e.* symmetric loading, smooth motion, etc.). After each 30-second effort, they rested for another 30 s before repeating the process again for a total of 10 rounds (Fig. 3a). We segmented the data into individual sitting and standing motions based on the measured chair force, the pelvis motion, and the knee kinematics.

We assessed the participants’ performance using several metrics: the number of cycles completed per round, average motion speeds, and inter-limb symmetry. Half cycles were counted if the participant had just completed the standing motion when time expired. For timing analysis, we isolated the specific motions and discarded any static time spent in the chair or at erect standing. We evaluated inter-limb symmetry in terms of ground reaction force (GRF) symmetry and knee torque symmetry. The GRF degree of asymmetry $DoA_\text {GRF}$ is defined as

1 $$\begin{aligned} DoA_\text {GRF} = (F_\text {b}- F_\text {p})/(F_\text {b}+ F_\text {p}) , \end{aligned}$$

where $F_\text {b}$ and $F_\text {p}$ are the vertical GRFs on the biological and prosthetic limbs, respectively. $DoA_\text {GRF}=0$ indicates perfect GRF symmetry, whereas $DoA_\text {GRF}=1$ and $DoA_\text {GRF}=-1$ indicate all force on the biological and prosthetic limbs, respectively. For knee moment symmetry, we utilized a slightly different symmetry index definition due to possible sign differences in the values of biological and prosthesis knee torques ($\tau _\text {b}$ and $\tau _\text {p}$, respectively). The knee torque degree of asymmetry, $DoA_\tau$, is defined as

2 $$\begin{aligned} DoA_\tau = (\tau _\text {b} - \tau _\text {p})/\max (|\tau _\text {b}|, |\tau _\text {p}|). \end{aligned}$$

The interpretation of $DoA_\tau$ is similar, with $DoA_\tau = 0$ still indicating perfect symmetry. In the case of extension moments, negative and positive values indicate bias towards the biological and prosthetic joints, respectively. However, $DoA_\tau$ is not bounded between $\pm 1$. Finally, we analyzed the average number of cycles performed per round across participants in each condition to evaluate performance changes as fatigue accumulated.

#### Incremental shuttle walk test

Next, we assessed the users’ ability to navigate combined sitting, standing, and walking tasks over a range of intensities in an incremental shuttle walk test. This test, which we first presented in [20], is based on fitness tests [25, 26] and required participants to repeatedly walk a circuit between two stools, each 45 cm tall and positioned approximately 6 m apart (Fig. 3b). Upon an auditory cue, participants stood up from a stool, walked to the opposite stool, turned around, and sat down to wait until the next cue. The cues were automated and followed this schedule: four cues at 65-second intervals, six at 43.3-second intervals, and eight at 32.5-second intervals, and so on, gradually increasing the minimum average walking pace from 0.1 m/s to 0.15 m/s and 0.2 m/s, respectively. The number of repetitions was chosen such that the total time at each pace was 260 s. Participants were allowed to choose their walking speed, but needed to reach the opposite stool before the next cue. The test ended if a participant failed to reach the stool on time for two consecutive laps.

We segmented the moving portions of each lap into sit/stand, walking, and transitioning categories based on kinematics and ground reaction force data. Transitioning was defined as periods when the pelvis center was within 1.0 m of the stools and the participant was not actively performing sitting or standing motions. This included the time required to stop walking and turn around in front of the stools prior to sitting down, as well as the time to begin walking after fully standing up. We evaluated the participants on the number of laps completed, the relative timing of each activity (sitting/standing, walking, and transitioning), their inter-limb symmetry, and their joint kinematics and kinetics. We also compared the minimum swing toe clearance during swing between conditions, which we calculated based on the minimum position of the 1st and 5th metatarsal markers during mid-swing.

#### Fast treadmill test

Finally, we investigated the participants’ ability to walk quickly in a self-paced treadmill test. We asked them to “walk as far as you can in fifteen minutes,” requiring them to maximize their speed while conserving enough energy to complete the test (Fig. 3c). We calculated each participant’s average selected treadmill speed for each stride for each condition, as well as the distance that they walked during the test. We evaluated the participants on their selected walking speed and their gait kinematics and kinetics. When comparing differences in peak kinetic values across conditions, we normalized the values by each stride’s average walking speed to account for speed differences across the different conditions, as moment magnitude often correlates with walking speed [27, 28]. Finally, we evaluated participants on the distance walked during the test, though prosthesis and treadmill balance limitations prevented some participants from completing the full fifteen minutes.

#### Data processing and statistical modeling

During the three tests, motion capture and ground reaction force data were recorded (Vicon, Oxford, UK). These data were processed in OpenSim 4.5 (Stanford, CA, USA) using the model from Rajagopal et al. [29] with appropriate modifications made to the prosthetic side inertial properties, similar to [30]. We report all joint angles and moments as positive in flexion/dorsiflexion. Force plates were located at each stool and in the treadmill, allowing individual measurements of each leg’s ground reaction forces and subsequent calculation of inverse dynamics. The walkway was not instrumented, and thus no walking joint kinetics were calculated for the incremental shuttle walk test.

To examine whether the prosthesis condition had a statistically significant effect on the biomechanical measures of interest, we utilized linear mixed-effects models (LMMs). LMMs were chosen for their ability to simultaneously account for both fixed effects (prosthesis condition [HKIC, ÖSSR, and PRES] and user mobility level [Higher (K4) and Lower (K3)]) and random effects (participant-specific random intercepts), thereby controlling for inter-participant variability. Interaction terms between mobility level and prosthesis condition were included to assess the factors within each mobility group. Technical replicates were averaged within each participant and condition to yield 21 independent observations per outcome.

For each outcome, we tested the null hypothesis that the prosthesis condition did not significantly affect the biomechanical measures within each mobility group. Model significance was assessed using a likelihood ratio test, comparing the full LMM (with all fixed effects and interactions) to a nested null model containing only an intercept and a participant random effect. To control for Type I error arising from assessing multiple outcomes, we grouped the outcomes into three primary functional domains: walking biomechanics, sit/stand biomechanics, and speed/endurance. We then applied a Bonferroni correction within each domain, such that outcomes were considered statistically significant only if they yielded $p < 0.05/n$, where *n* is the number of outcomes analyzed within each functional domain ($n=4$ for sit/stand, $n=8$ for walking, and $n=9$ for speed/endurance). Outcomes meeting this criterion were subjected to follow-up analyses to further investigate condition-specific effects.

In most plots below, we provide boxplots of each participant’s individual data as well as the results of the best-fit LMMs. For clarity in the data with high numbers of samples, we omit the outlier markers from the boxplots, specifically the samples outside of $[Q1-1.5\times IQR, Q3+1.5\times IQR]$. We indicate statistical significance of the fixed effects in the plots with asterisks: * $\iff 0.05 > p \ge 0.01$, ** $\iff 0.01 > p \ge 0.001$, and *** $\iff 0.001 > p$. All fixed effect results are given as changes relative to the model’s estimated PRES baseline for each mobility level. Significance marks above a single condition indicate significant differences from the baseline, whereas significance marks between conditions indicate significant contrasts.

#### Qualitative participant feedback

Finally, after the conclusion of their three testing days, we asked each participant for qualitative feedback regarding their experience with each condition in the form of an informal exit video interview (see Additional file 2). We prompted the participants to describe the differences they perceived between the conditions, specifically relating to the ease of sitting, standing, and walking. We began by asking about their prescribed passive prostheses compared to the Power Knee in general, and then we asked them to compare the two Power Knee control policies. While it was impossible to blind the participants between the PRES condition and the two Power Knee conditions, we importantly did not tell them which control policy was used on which day until after the video interview. Two research team members independently reviewed the video interview footage, noting common themes and important perspectives from each participant. Note that participant TF01 had participated in a prior pilot study, and thus was able to discern for himself which control policy was which. Therefore, we did not include his qualitative feedback in our overall findings regarding the differences between the HKIC and ÖSSR conditions, as it may have been biased. Participant TF04 was unable to provide qualitative feedback on the PRES condition due to scheduling constraints.

## Results

### Repeated sit/stand test

**Fig. 4:**
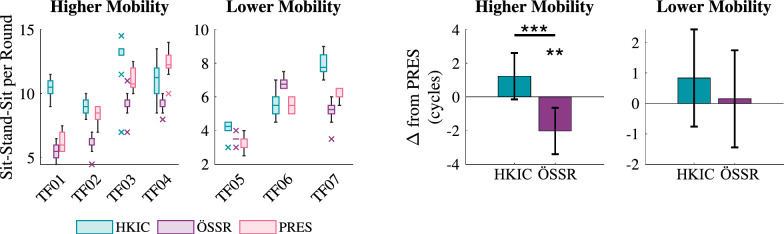
Left: Boxplots of the number of sit-stand-sit cycles completed in each round of the repeated rapid sit/stand test for each participant and condition. Right: The estimated changes in cycle count due to each condition from the PRES baseline for each mobility level based on the best-fit LMM of the data. Error bars indicate 95% confidence intervals

Figure 4 shows the number of sit/stand cycles completed under each prosthesis condition in the repeated sit/stand test, where participants attempted to complete as many cycles as possible within the allotted time. The effect of the prosthesis condition varied between the two mobility groups. For the higher mobility group, the best-fit LMM ($p<0.001$) indicated that relative to the PRES case, the ÖSSR condition produced $2.0\pm 1.4$ fewer cycles ($p=0.007$), whereas the HKIC condition did not produce a statistically significant difference ($1.2\pm 1.4$ more cycles, $p=0.078$). The contrast between HKIC and ÖSSR was significant for the higher mobility group ($p<0.001$). No significant effects between the different prosthesis conditions were observed in the lower mobility group (HKIC: $p=0.268$, ÖSSR: $p=0.848$, contrast: $p=0.375$).

**Fig. 5:**
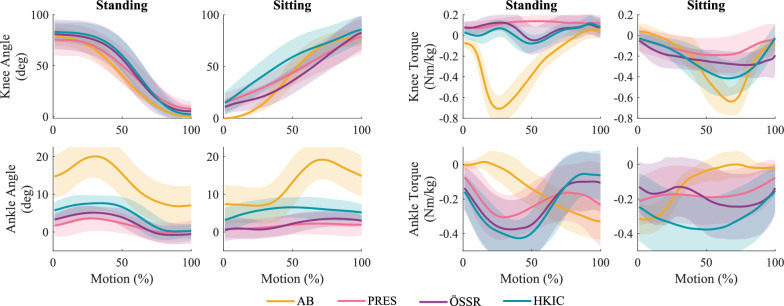
Inter-participant average knee and ankle kinematics and kinetics (prosthetic side) during the rapid repeated sit/stand test produced in the prescribed passive condition (PRES), the phase-based controller condition (HKIC), and the default controller condition (ÖSSR). Able-bodied trajectories (AB) from [31] are shown as well for reference. Shaded regions represent $\pm 1$ standard deviation

The inter-participant mean kinematics and kinetics for the individual standing and sitting motions are shown in Fig. 5. In terms of knee kinematics, all three conditions appeared quite similar, though the HKIC knee trajectory differed from the others during the first half of the sitting motion. Larger differences in the knee’s kinetic behavior were observed, particularly in sitting where the HKIC condition more closely matched the shape and the peak magnitude of the reference able-bodied behavior. In standing, all conditions’ torque profiles were much smaller than typical able-bodied levels. The ankle kinematics and kinetics also differed substantially from able-bodied behavior in all conditions due to the constraints of passive prosthetic feet.

**Fig. 6:**
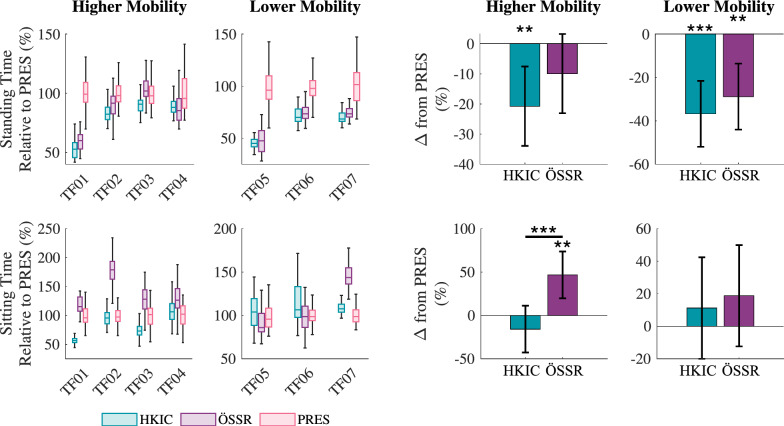
Left: Boxplots of the time each participant spent standing (top row) and sitting (bottom row) under each condition during the repeated rapid sit/stand test. Values are normalized to each participant’s average time in the PRES condition (which is still shown to give distribution information). Note that these timings only include the duration of the specific motions, excluding the time spent statically sitting or standing between motions. Right: The estimated changes in standing and sitting timing due to each condition from the PRES baseline for each mobility level based on the best-fit LMM of the data. Error bars indicate 95% confidence intervals.

Figure 6 shows the time required to complete the standing and sitting motions for each participant under each condition, normalized to their average time for each motion in the PRES condition. The best-fit LMM was statistically significant relative to the null model ($p<0.001$). In the higher-mobility group, the HKIC condition reduced the standing time by $-20.8\pm 13.1$% ($p=0.004$). The ÖSSR condition did not show a statistically significant difference in standing time ($-9.5\pm 13.1$%, $p=0.128$). The effect difference between HKIC and ÖSSR was also not significant ($p=0.100$). For the lower mobility group, both Power Knee conditions produced larger reductions in standing time. The HKIC condition reduced it by $36.7\pm 15.1$% ($p<0.001$) and the ÖSSR condition reduced it by $28.8\pm 15.1$% ($p=0.001$). The contrast between HKIC and ÖSSR was not significant ($p=0.283$).

In sitting, the LMM ($p=0.005$) likewise indicated that the HKIC condition did not significantly affect the motion time for the higher mobility participants ($-15.8\pm 27.0$%, $p=0.233$). In contrast, the ÖSSR condition significantly increased the sitting time by $46.9\pm 27.0$% ($p=0.002$). The contrast between HKIC and ÖSSR was also significant ($p<0.001$). However, the lower mobility group did not show significant differences in sitting time between any of the prosthesis conditions (HKIC: $p=0.446$, ÖSSR: $p=0.351$, contrast: $p=0.613$).

**Fig. 7:**
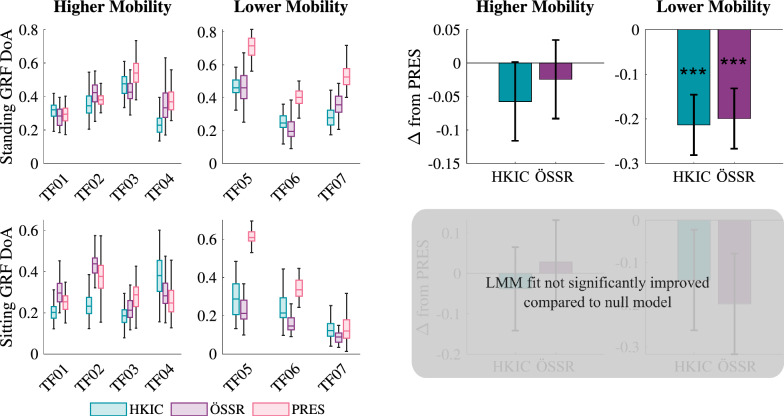
Left: Boxplots of the average GRF degree of asymmetry ($DoA_\text {GRF}$) for each participant and condition during the repeated rapid sit/stand test. More positive values indicate favoring the biological limb, while more negative values indicate favoring the prosthetic limb. Zero indicates perfect symmetry. Right: The estimated changes in GRF DoA due to each condition from the PRES baseline for each mobility level based on the best-fit LMM of the data. For the sitting data, the best-fit LMM did not show a statistically significant improvement over a null model ($p=0.057$). Error bars indicate 95% confidence intervals

The ability to symmetrically load the limbs during both standing and sitting varied substantially across participants as seen in Fig. 7, where $DoA_\text {GRF}=0$ indicates perfect GRF symmetry, positive values favor the biological limb, and negative values favor the prosthetic limb. In standing with the higher mobility group, the LMM ($p<0.001$) indicated that the HKIC and ÖSSR conditions did not have a statistically significant effect on GRF symmetry (HKIC: $-0.058 \pm 0.059$, $p=0.054$; ÖSSR :$-0.024\pm 0.059$, $p=0.390$). The difference between the HKIC and ÖSSR conditions was not significant ($p=0.246$). In contrast, the lower mobility group displayed a stronger response to the prosthesis conditions, with the HKIC condition reducing asymmetry by $-0.214\pm 0.068$ ($p<0.001$) and the ÖSSR condition reducing it by $-0.199\pm 0.068$ ($p<0.001$). No significant difference was detected between the HKIC and ÖSSR conditions for the lower mobility group ($p=0.661$).

In sitting, some strong within-participant effects of the prosthesis condition were observed, such as the large reductions in asymmetry by the Power Knee conditions compared to the PRES condition for participant TF05. However, other participants showed less of an effect (e.g., TF07), and no clear between-participant trends emerged. Compared to the null model, the best-fit LMM did not provide a statistically significant improvement in fit ($p=0.057$).

**Fig. 8:**
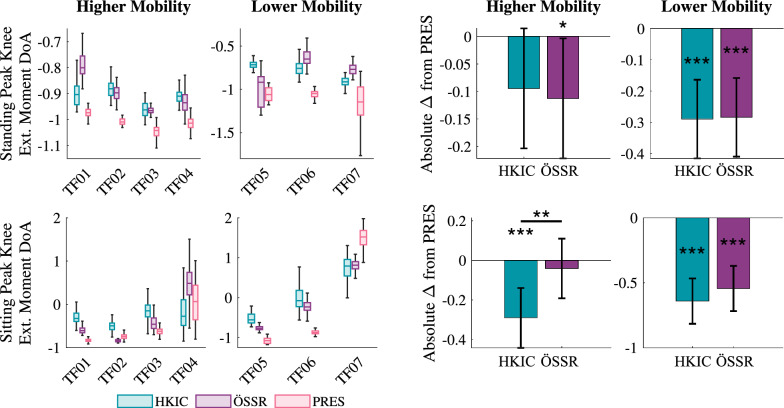
Left: Boxplots of the peak knee extension moment degree of asymmetry ($DoA_\tau$) for each participant and condition during the repeated rapid sit/stand test. In the case of extension moments (which are predominately negative), more positive values indicate favoring the prosthetic limb, while more negative values indicate favoring the biological limb. Zero indicates perfect symmetry. Right: The estimated changes in the absolute peak knee extension moment $DoA_\tau$ due to each condition from the PRES baseline for each mobility level based on the best-fit LMM of the data. More negative values indicate better symmetry improvements. Error bars indicate 95% confidence intervals.

The symmetry in the peak knee extension torque generally improved in the two Power Knee conditions compared to the PRES condition (Fig. 8). Recall that $DoA_\tau = 0$ corresponds to perfect symmetry, while more negative values indicate favoring the biological limb. In standing with the higher mobility group, the LMM fit ($p<0.001$) to the magnitude of peak knee moment asymmetry, $|DoA_\tau |$, indicated an improvement of $-0.113\pm 0.109$ ($p=0.043$) for the ÖSSR condition but no significant change for the HKIC condition ($-0.095\pm 0.109$, $p=0.084$). The contrast between HKIC and ÖSSR was small and insignificant ($p=0.726$). Larger and more significant effects were observed in the lower mobility group, where the HKIC condition reduced peak knee moment asymmetry by $-0.290\pm 0.126$ ($p<0.001$), and the ÖSSR condition reduced it by $-0.284\pm 0.126$ ($p<0.001$). The contrast between HKIC and ÖSSR was again insignificant ($p=0.924$).

In sitting, some participants had larger peak knee extension moments on the prosthetic side, others had larger ones on the biological side, and some were fairly symmetric. This was the only sit/stand symmetry metric where participants did not show a clear bias towards one leg. In the higher mobility group, the best-fit LMM ($p<0.001$) indicated that the HKIC condition produced a meaningful reduction in $|DoA_\tau |$ of $-0.290\pm 0.152$ ($p=0.001$), while the ÖSSR condition did not have a significant effect ($-0.041\pm 0.152$, $p=0.573$). The contrast between HKIC and ÖSSR was significant ($p=0.003$). For the lower mobility group, both Power Knee conditions created large asymmetry reductions. The HKIC improved peak knee extension moment symmetry by $0.641\pm 0.174$ ($p<0.001$) and the ÖSSR condition improved it by $0.544\pm 0.174$ ($p<0.001$). The contrast between HKIC and ÖSSR was insignificant for the lower mobility group ($p=0.254$).

**Fig. 9:**
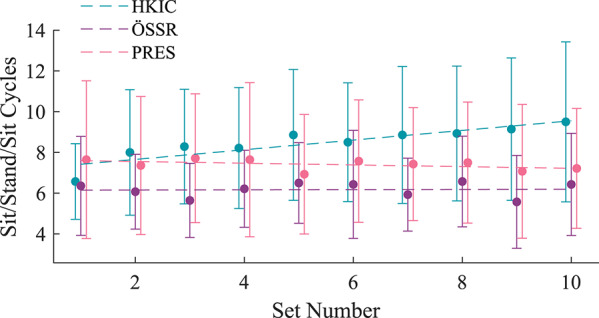
Solid circles indicate the inter-subject average number of sit/stand/sit cycles completed in each set of the repeated sit/stand experiment, separated by condition. The dashed lines show the predicted means from the linear model. Error bars indicate $\pm 1$ standard deviation

The inter-participant average sit/stand/sit cycle counts from each set of the rapid repeated sit/stand test under each condition are shown in Fig. 9. The LMM fit to the data with an interaction term between set number and condition ($p<0.001$) showed that both the PRES and ÖSSR conditions showed no significant change in completion count over time ($p=0.341$ and $p=0.267$, respectively). However, the HKIC condition showed an increase in completion count at the rate of $0.25 \pm 0.14$ cycles per set ($p<0.001$).

### Incremental shuttle walk test

**Fig. 10:**
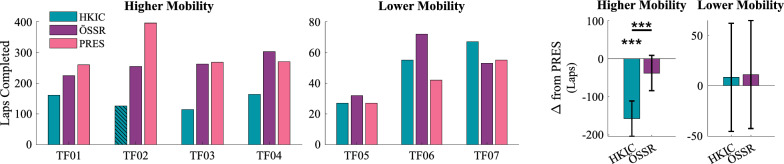
Left: The number of laps completed during the incremental shuttle walk test under each prosthesis condition. Participants are separated into higher and lower mobility groups for scale. The bar for TF02 in the HKIC condition is hatched because the participant had to stop the test early at lap 126 due to a thermal issue with the Power Knee, rather than due to his own physical limitations. Right: The estimated changes in lap count from the PRES baseline due to each condition in each mobility group based on the best-fit LMM. Error bars indicate 95% confidence intervals

The effects of the different conditions on the number of laps completed in the incremental shuttle walk test varied between the higher and lower mobility groups (Fig. 10). The LMM ($p<0.001$) indicated that in the higher mobility groups, the HKIC condition reduced the number of laps completed by $-157.3\pm 46.3$ ($p<0.001$), while the ÖSSR condition did not significantly reduce it ($-37.8\pm 46.3$, $p=0.103$). The contrast between HKIC and ÖSSR was significant ($p<0.001$). In the lower mobility group however, the two Power Knee conditions did not have a significant effect on the lap completion count (HKIC: $p=0.744$, ÖSSR: $p=0.667$, contrast: $p=0.917$). Note that TF02 was unable to complete the experiment with the HKIC condition due to a thermal problem with the Power Knee during lap 126.

**Fig. 11:**
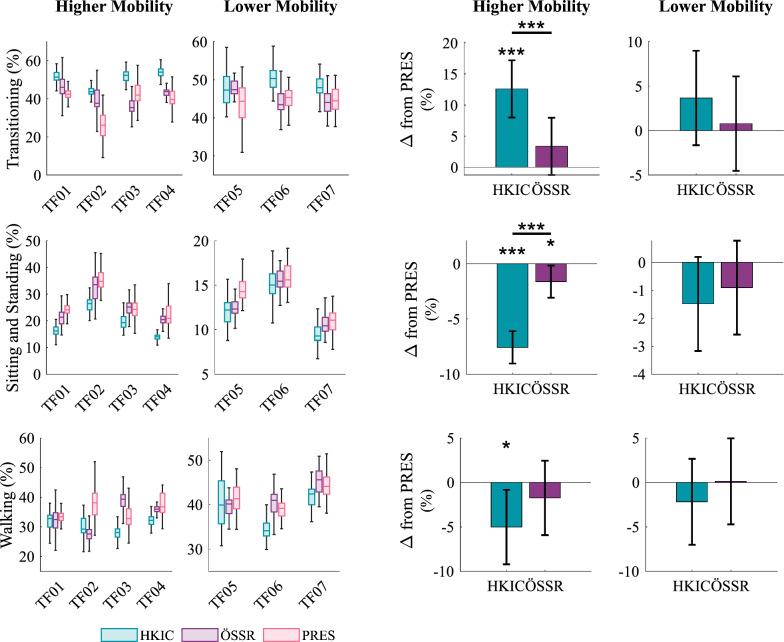
Left: Boxplots of the amount of time each participant spent transitioning (top), sitting and standing (middle), and walking (bottom) during the incremental shuttle walk test as a percentage of each lap’s moving duration. Right: The estimated changes in percentage from the PRES baseline caused by each condition for each mobility group based on the best-fit LMM. Error bars indicate 95% confidence intervals.

The proportion of time spent performing the different activities within each lap varied across conditions and mobility groups (Fig. 11). The best-fit LMMs for transitioning ($p=0.001$), sit/stand ($p<0.001$), and walking ($p=0.004$) were all statistically significant relative to the null model. For the higher mobility group, transitions in the HKIC condition comprised a larger portion of the participants’ lap time compared to both the PRES ($12.59\pm 4.60$% more, $p<0.001$) and ÖSSR conditions (9.23% more, $p<0.001$). No significant difference was observed for the ÖSSR condition compared to PRES ($3.35\pm 4.60$% more ($p=0.141$). For the lower mobility group, no significant differences were observed (HKIC: $p=0.163$, ÖSSR: $p=0.760$, contrast: $p=0.265$). In contrast, sit/stand motions comprised the lowest percentage of time with the HKIC condition compared to the PRES condition ($7.57\pm 1.46$% less, $p<0.001$) for the higher mobility group. The ÖSSR condition similarly reduced sit/stand percentages, but only by $1.62\pm 1.46$% ($p=0.032$). The decrease in HKIC relative to ÖSSR was significant ($p<0.001$). The lower mobility group did not display significant differences in sit/stand percentages with the two Power Knee conditions (HKIC: $p=0.079$, ÖSSR: $p=0.268$, contrast: $p=0.474$). The walking results were similar to the sit/stand results, with the participants spending a lower proportion of their time walking in the HKIC condition compared to the PRES condition ($5.02\pm 4.19$% less, $p=0.022$). No meaningful difference was observed for the ÖSSR condition relative to PRES ($1.73\pm 4.19$% less, $p=0.391$), and the contrast between HKIC and ÖSSR did not reach significance ($p=0.116$). No significant differences were observed for the lower mobility group (HKIC: $p=0.353$, ÖSSR: $p=0.955$, contrast: $p=0.326$).

**Fig. 12:**
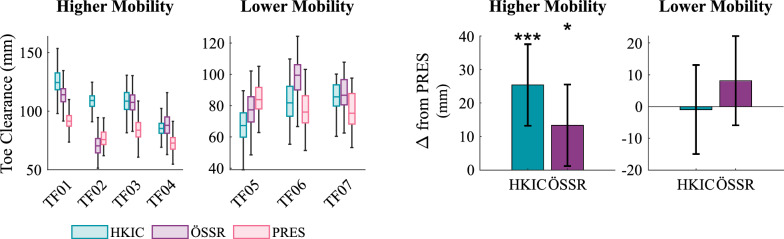
Left: Boxplots of the minimum swing toe clearance during the incremental shuttle walk test for each participant and condition. Right: The estimated changes in toe clearance from the PRES baseline caused by each condition for each mobility group based on the best-fit LMM. Error bars indicate 95% confidence intervals.

During the walking portions of the experiment, the Power Knee conditions showed improvements in various kinematic features. The best fit LMM ($p=0.006$) indicated that the minimum toe clearance (Fig. 12), which is important to avoid toe stubbing, was increased for the higher mobility group with the Power Knee relative to the PRES condition by $25.4\pm 12.2$ mm in the HKIC condition ($p<0.001$) and by $13.4\pm 12.2$ mm in the ÖSSR condition ($p=0.033$). The contrast between HKIC and ÖSSR did not reach statistical significance ($p=0.052$). In contrast, no clinically or statistically significant changes in toe clearance were observed in the lower mobility group (HKIC: $p=0.888$, ÖSSR: $p=0.235$, contrast: $p=0.188$).

**Fig. 13:**
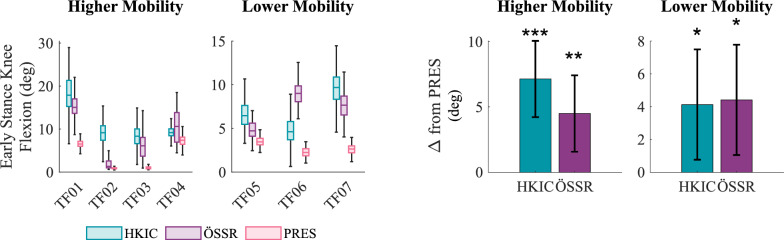
Left: Boxplots of the peak early stance knee flexion angle during the incremental shuttle walk test for each participant and condition. Right: The estimated changes in early stance knee flexion from the PRES baseline caused by each condition for each mobility group based on the best-fit LMM. Error bars indicate 95% confidence intervals.

Likewise, early stance knee flexion increased in the Power Knee conditions for both mobility groups ($p=0.002$, Fig. 13). Compared to the PRES condition in the higher mobility group, the HKIC condition increased early stance flexion by $7.1\pm 2.9$ deg ($p<0.001$) and the ÖSSR condition increased it by $4.5\pm 2.9$ deg ($p=0.005$). The contrast between the HKIC and ÖSSR conditions was not statistically significant ($p=0.073$). The lower mobility group likewise observed increases in early stance knee flexion, with the HKIC condition increasing it by $4.1\pm 3.4$ deg ($p=0.020$) and the ÖSSR condition increasing it by $4.4\pm 3.4$ deg ($p=0.014$). The contrast between HKIC and ÖSSR was not significant ($p=0.858$).

**Fig. 14:**
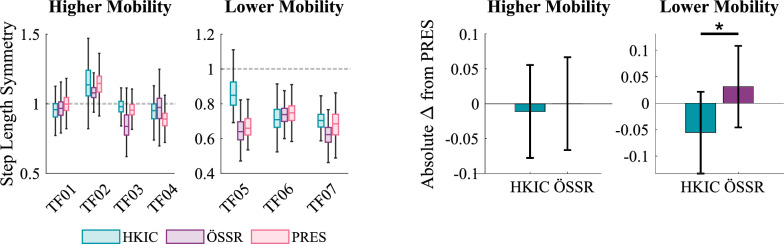
Left: Boxplots of the step length symmetry index for each participant and condition during the incremental shuttle walk test. A step length symmetry index of 1 corresponds to perfectly symmetric step lengths, while values greater than 1 indicate longer steps with the prosthesis. Right: The estimated changes in absolute step length asymmetry caused by each condition for each mobility group based on the best-fit LMM. More negative values indicate better symmetry improvements, and error bars indicate 95% confidence intervals.

Step length symmetry, defined as the ratio between the prosthetic and the subsequent biological leg step lengths, showed mixed, participant-dependent results. It was either improved (TF04 and TF05) or unaffected by the HKIC condition relative to the PRES condition (Fig. 14). For some participants, the ÖSSR condition improved step length symmetry (TF02), whereas this condition worsened symmetry for others (TF03). On average, the best-fit LMM ($p<0.001$) did not indicate a significant relationship between the conditions and step length symmetry for either mobility group. The only statistically significant effect was the small decrease in absolute asymmetry of $-$0.087 in the HKIC condition compared to the ÖSSR condition in the lower mobility group ($p=0.029$).

### Fast treadmill test

**Fig. 15:**
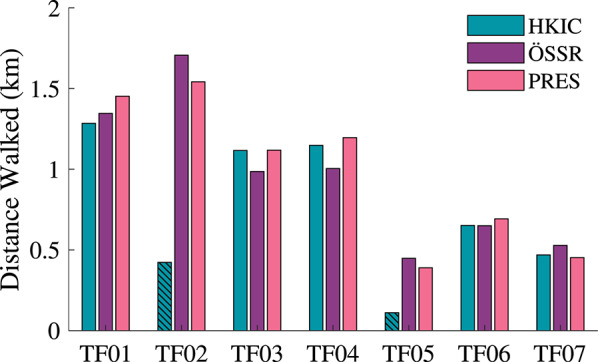
The distance each participant walked during the fast treadmill test under each condition. The HKIC condition’s results for Participants TF02 and TF05 are shown with hatched bars, as they did not complete full 15 min of the experiment.

The ability to complete the fast treadmill test varied across participants, and the distance walked by each participant is shown in Fig. 15. For some participants, especially the lower mobility ones, walking on a treadmill was more difficult than walking over-ground. Some of the lower mobility participants found the HKIC condition most difficult on the treadmill, as its more dynamic behavior was furthest from what they were accustomed to with their PRES device. We believe that the increased cognitive burden of learning a new, dynamic control policy, as well as the lack of optic flow feedback, likely contributed to this perceived difficulty. Specifically, participant TF05 chose not to complete the HKIC condition because he felt that it was too difficult to walk safely on the treadmill. TF02 was also unable to complete the HKIC condition, but instead due to thermal overload on the Power Knee at his desired fast walking pace ($\approx$ 1.93 m/s).

**Fig. 16:**
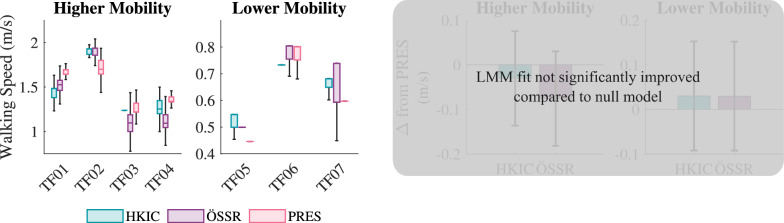
Left: Boxplots of the treadmill speed selected by each participant for each condition during the fast walking test. Right: The best-fit LMM was not statistically significant after Bonferroni correction ($p=0.018$).

Minimal differences were observed in the participants’ selected walking speed across conditions (Fig. 16). In multiple trials with the lower mobility group, the participants elected not to change the treadmill speed much at all after the initial selection. After Bonferroni correction, the likelihood ratio test showed that the LMM was not a significantly better predictor of walking speed than the null model ($p=0.018$).

**Fig. 17:**
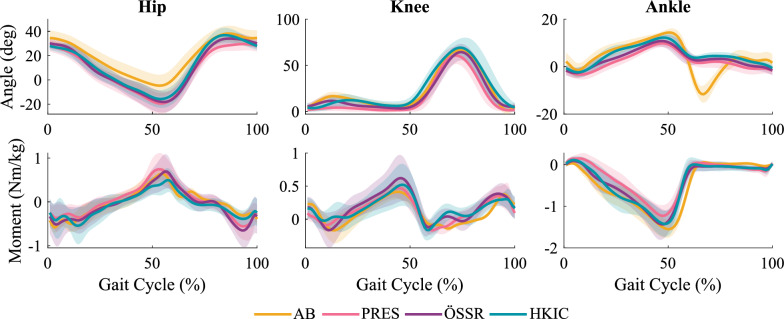
Inter-participant average hip, knee, and ankle kinematics and kinetics (prosthetic side) for the fast treadmill tests under each condition. For comparison, average able-bodied kinematics and kinetics for 1.0 m/s level walking are included from [32]. Shaded regions represent $\pm 1$ standard deviation

**Fig. 18:**
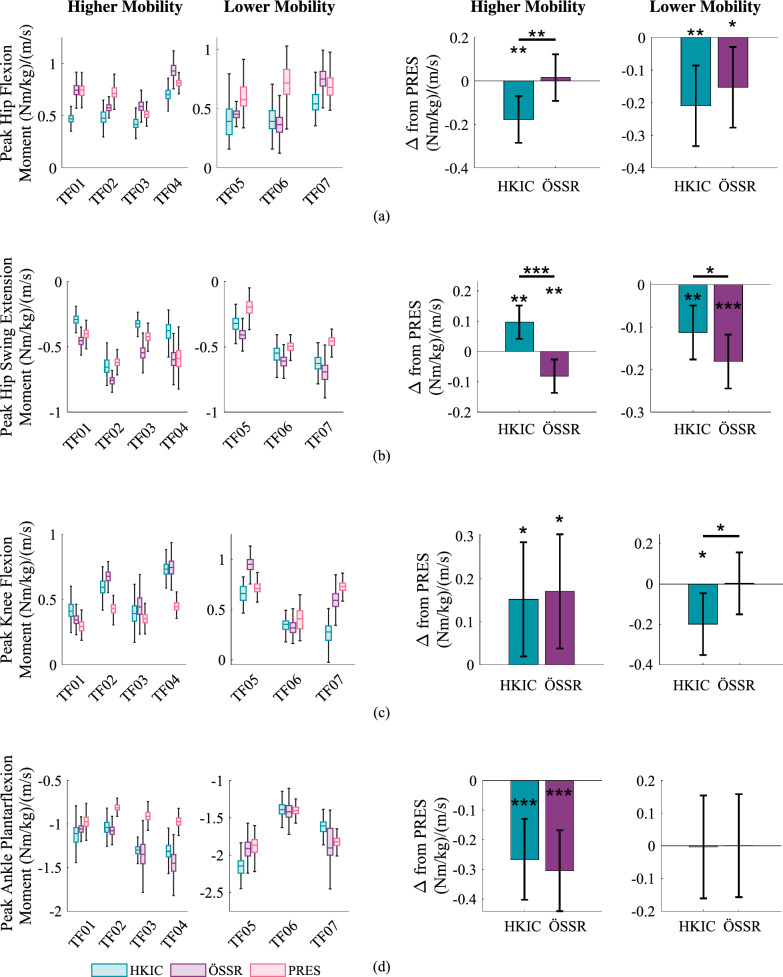
Left: Boxplots of the relevant kinetic features on the prosthetic side during the fast 15 min walk test, normalized by walking speed. These include **a** the peak swing hip flexion moment, **b** the peak swing hip extension moment, **c** the peak knee stance flexion moment, and **d** the peak ankle plantarflexion moment. Right: The estimated changes in the kinetic features caused by each condition for each mobility group based on the best-fit LMM. For **a** and **c**, negative values indicate decreases in moment magnitude, while for **b** and **d**, positive values indicate decreases in moment magnitude. Error bars indicate 95% confidence intervals

**Fig. 19:**
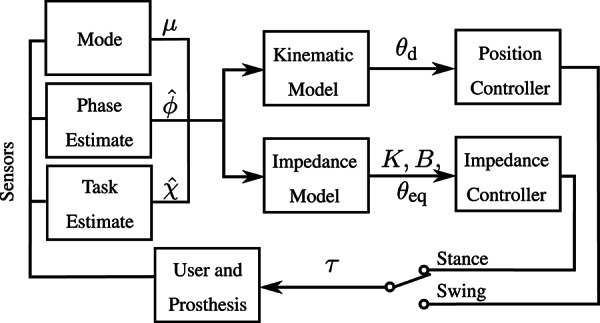
A block diagram of the Hybrid Kinematic Impedance Controller used in this work. Real-time estimates of gait phase $\hat{\phi }$, task variation $\hat{\chi }$ (*i.e.* walking speed), and control mode $\mu$ (*i.e.* Walk vs. Sit/Stand) define desired joint impedance parameters $K, B, \theta _\text {eq}$ and joint angles $\theta _\text {d}$ using data-driven models. The torque commands $\tau$ are calculated using an impedance controller during stance or a position controller during swing

Despite the lack of sensitivity to the condition in the distance and speed metrics, some more substantial differences were observed in the joint kinetics. The inter-participant average trajectories shown in Fig. 17 give a rough idea of the mean biomechanical behavior of each condition, but they should not be over-interpreted because speed was not held consistent across conditions and participants. The peak swing hip flexion moment (*i.e.* pull-off moment) on the prosthetic side was reduced in the HKIC condition compared to the PRES condition (Fig. 18a). The LMM ($p=0.003$) indicated that in the higher mobility group, the HKIC condition reduced the peak hip flexion torque requirement on average by $-0.18\pm 0.11$ (Nm/kg)/(m/s) ($p=0.003$), while the ÖSSR condition did not display a statistically significant effect ($0.02\pm 0.11$ (Nm/kg)/(m/s), $p=0.767$). The contrast between HKIC and ÖSSR was significant ($p=0.002$). For the lower mobility groups, both Power Knee conditions reduced the peak hip flexion moment by an even larger amount. The HKIC condition reduced it by $-0.21\pm 0.12$ (Nm/kg)/(m/s) ($p=0.003$) and the ÖSSR condition reduced it by $-0.15\pm 0.12$ (Nm/kg)/(m/s) ($p=0.019$). The contrast between HKIC and ÖSSR was not significant for the lower mobility group ($p=0.343$).

Likewise, the peak hip extension moment in late swing (*i.e.* hip retraction moment) was also affected by the prosthesis condition (Fig. 18b). The best-fit LMM ($p<0.001$) indicated that in the higher mobility group, the HKIC condition reduced the normalized peak hip extension moment magnitude by $-0.10\pm 0.06$ (Nm/kg)/(m/s) ($p=0.002$), and the ÖSSR condition increased it by $0.08\pm 0.06$ (Nm/kg)/(m/s) ($p=0.007$). The contrast between the HKIC condition and the ÖSSR condition was significant ($p<0.001$). In the lower mobility group, both the HKIC and ÖSSR conditions increased the peak hip extension moment magnitude (HKIC: $0.11\pm 0.06$ (Nm/kg)/(m/s), $p=0.002$, ÖSSR: $0.18\pm 0.06$ (Nm/kg)/(m/s), $p<0.001$). The HKIC condition increased it by a smaller amount than the ÖSSR condition ($p=0.037$).

The higher mobility participants also demonstrated increases in the normalized peak stance knee flexion moment (occurring between mid-stance and toe-off) in the two Power Knee conditions ($p=0.006$, Fig. 18c). The HKIC condition increased it by $0.15\pm 0.13$ (Nm/kg)/(m/s) ($p=0.028$) and the ÖSSR condition increased it by $0.17\pm 0.13$ (Nm/kg)/(m/s) ($p=0.015$). The contrast between the HKIC and ÖSSR conditions was not significant ($p=0.770$). For the lower mobility group, the HKIC decreased the peak stance knee flexion moment by $-0.19\pm 0.15$ (Nm/kg)/(m/s) ($p=0.015$), and the ÖSSR condition had no significant effect ($p=0.959$). The contrast between HKIC and ÖSSR was significant for the lower mobility group ($p=0.013$). No statistically or clinically significant differences were observed between conditions in the peak knee extension moment at weight acceptance ($p=0.369$).

Finally, participants demonstrated increased peak ankle plantarflexion moment magnitudes (*i.e.* push-off moments) on the prosthetic side with the Power Knee conditions ($p<0.001$), specifically in the higher mobility group (Fig. 18d). The HKIC condition increased the peak magnitude by $0.27\pm 0.14$ (Nm/kg)/(m/s) ($p<0.001$) and the ÖSSR condition increased it by $0.30\pm 0.14$ (Nm/kg)/(m/s) ($p<0.001$). The contrast between the HKIC and ÖSSR conditions was not significant ($p=0.558$). No change was observed in the lower mobility group in either condition (HKIC: $p=0.965$, ÖSSR: $p=0.994$, contrast: $p=0.958$).

### Qualitative participant feedback

In the post-experiment interviews, all participants mentioned that they could perceive substantial differences between the Power Knee conditions and their prescribed passive prostheses for the activities tested. Many participants felt that the Power Knee provided meaningful assistance (TF01, TF02, TF05, TF06, TF07), and some felt that they were less sore following the experiments with it compared to their passive knees (TF02, TF07). Many participants specifically noted reduced effort in sit-to-stand transitions with the Power Knee compared to their passive knees (TF01, TF02, TF06, TF07). Perceptions of differences in walking were more varied between participants, with some noting that it felt easier with the Power Knee (TF01, TF06, TF07) while others did not mention large differences in walking. TF05 felt that the Power Knee forced him to take larger steps, which he felt could be undesirable in certain conditions like walking in a crowd. The participants also noted some perceived downsides of the Power Knee. Participant TF01 felt that the Power Knee’s increased weight was undesirable during walking, while TF07 reported that they did not notice the added weight. The other participants did not mention the prosthesis weight in their feedback. Participant TF02 also felt that he was less agile with the Power Knee compared to his passive prosthesis, and that while it would be okay for day-to-day activities, it may limit him in faster-paced activities. Finally, participant TF04 noted that both Power Knee conditions had high learning curves that made it more difficult to perform the activities to the best of her ability without more practice.

The participants also perceived notable differences between the Power Knee conditions under the ÖSSR and HKIC control policies. Many (TF02, TF03, TF04, TF06, TF07) reported that the HKIC control policy was more difficult to learn how to use compared to the ÖSSR control policy. Many felt that the ÖSSR control policy’s behaviors were more similar to those of their passive MPKs compared to those of the HKIC policy. However, many also reported that, after learning how to use it, the HKIC control policy provided them with more assistance and produced smoother, more natural behaviors (TF02, TF03, TF04, TF06, TF07). TF04 in particular mentioned that the ÖSSR condition “felt like a heavier version of [her] X3” while walking, whereas the HKIC condition required less effort during walking. Finally, multiple participants (TF04, TF06) noted that walk-to-sit transitions were slower and required more attention in the HKIC condition compared to the ÖSSR condition.

## Discussion

### Clinical effects of the Power Knee relative to passive knees

Our results showcase multiple areas where the Power Knee produced clear clinical effects, some positive and some negative, compared to the users’ prescribed passive knees. Notably, the impact of the Power Knee often depended on the user’s mobility level, although some effects were observed across both higher and lower mobility groups.

#### Sit/Stand

The Power Knee most consistently affected sit/stand in the lower mobility group. Compared to their prescribed passive prostheses, the lower mobility participants displayed a reduction in standing time (Fig. 6), an improvement in standing GRF symmetry (Fig. 7), and an improvement in knee torque symmetry during both sitting and standing (Fig. 8). This improved sit/stand symmetry may potentially help the lower mobility group mitigate overuse injuries and lower back pain over the long-term, as asymmetric loading has been suggested as a potential cause of degradation and osteoarthritis in the intact joints [33, 34]. While less consistent, some effects were still observed in the higher mobility group, such as improvements in sitting knee torque symmetry (Fig. 8) and standing speed (Fig. 6) in the HKIC condition. The discrepancy in benefits could be explained by strength differences between the mobility groups, where the higher mobility group was more able to complete the task without having to rely on assistance from the Power Knee.

Further, the standing speed improvements for the lower mobility group in both powered conditions suggest that the standing task was easier for them with the Power Knee compared to the PRES condition, as the participants’ goal was to complete as many sit/stand cycles as possible during the experiment. This finding differs from that of [14], who reported no change in standing time with a previous Power Knee model relative to the C-Leg. The participants’ qualitative feedback also reflected a perceived reduction in standing difficulty with the Power Knee. Beyond time savings, a reduction in standing difficulty could have real clinical value, as healthy adults perform at least 45 sit-to-stand transfers per day on average [35, 36]. If standing from a chair remains challenging or fatiguing, prosthesis users may opt to perform the movement less frequently. Because higher frequencies of sit-to-stand activity are associated with maintained mobility and slower functional decline in older adults [37], interventions that make this movement less difficult may contribute to greater daily mobility and functional independence for prosthesis users.

However, not all speed-related effects with the Power Knee were positive. The sitting motions were slower in the ÖSSR condition compared to the PRES condition for the higher mobility group (Fig. 6), likely contributing to the lower average sit/stand cycle completion count per round (Fig. 4). These results highlight that while the Power Knee can help lower mobility users in sit/stand tasks, it may inhibit rapid and agile motion for higher mobility users, depending on the control policy used.

Interestingly, the knee torque applied by the Power Knee to achieve these symmetry and standing time improvements was not drastically different from the PRES condition (Fig. 5). While the inverse dynamics results show some small extension torques during standing in the Power Knee conditions, they were still far from the torque magnitudes observed in able-bodied standing motions. Our previous work has shown that this non-biomimetic torque profile is likely due to the kinematic limitations imposed by the passive ankle joint [20]. It is possible that a prosthesis with both a robotic knee and ankle that enables a more biomimetic knee torque profile could elicit even stronger sit/stand loading symmetry and standing speed benefits, as in [10]. Further, this result suggests that robotic prostheses with smaller and lighter motors may still be able to provide substantial user benefits despite their lower torque capabilities [38, 39].

#### Walking

The Power Knee’s primary effects during walking appeared in the participants’ kinematics and kinetics. The significant increases in early stance knee flexion (Fig. 13) that occurred under both control policies likely helped in absorbing heelstrike impacts and maintaining forward momentum, which may result in smoother and more natural walking gaits [40]. We also observed increased toe clearance in the Power Knee conditions with the higher mobility group. Toe clearance has strong clinical relevance, as tripping is a major source of falls for lower-limb prosthesis users [41]. The lower mobility group did not show this increased toe clearance, which may be because they typically chose to walk at speeds that were slower (mean 0.62 m/s) than the HKIC policy’s training data (minimum 0.80 m/s [7]). Future work should investigate if including more slow-speed walking data when training the HKIC control policy’s model could allow this benefit to transfer to the lower mobility group.

Kinetic walking benefits were also observed with the Power Knee. Although the Power Knee can only actuate the knee joint, the HKIC and ÖSSR conditions displayed higher peak plantarflexion torques compared to the passive knees in the higher mobility group (Fig. 18d). This plantarflexion torque is important in both accelerating the user’s center of mass forward as well as initiating appropriate swing dynamics [42]. Fig. 18c indicates this torque increase may have been achieved by providing higher knee flexion torques during mid-to-late stance, which increased the deflection and energy stored in the passive ankle/foot to enable a larger push-off torque resembling able-bodied profiles. The lower mobility group did not display this benefit, again potentially due to their slower selected walking speeds. In contrast, both the higher and the lower mobility groups displayed substantial peak hip flexion moment reductions in swing with the HKIC condition, as well as the lower mobility group with the ÖSSR condition (Fig. 18a). Increased hip flexor activity is a known asymmetric compensation in above-knee amputee populations that may contribute to lower back pain [43]. The increased push-off torque in the Power Knee conditions likely contributed to this reduction by providing the prosthesis with more anterior momentum at the time of toe-off, reducing the need to pull off with the prosthesis-side hip. It could also be due to the Power Knee’s ability to actively flex the knee during mid-swing, allowing the participants to achieve sufficient toe clearance without having to induce knee flexion with their residual hip. The HKIC condition also reduced the peak hip extension moment magnitude in late swing for the higher mobility group compared to the PRES condition (Fig. 18b). This retraction moment helps passive prosthesis users ensure that the knee is fully extended and ready for weight acceptance, and the phase-based synchronization of knee extension in the HKIC condition appears to have reduced this requirement. However, the lower mobility group did not display this benefit, perhaps again due to their slower walking speeds. The hip moment magnitude reductions may have contributed to the perceived reduction in walking effort reported by some participants (e.g., TF02, TF04).

#### User endurance and speed

Interestingly, these kinematic and kinetic improvements did not result in increased performance in terms of walking speed or laps completed for either mobility group. For the higher mobility group in particular, the HKIC condition reduced participants’ performance in the incremental shuttle walk test (Fig. 10), likely due to its slower walk-to-sit transitions. While this condition increased standing speed and reduced the walking hip effort, these benefits appeared to be outweighed by the reduced transition speed. Less of a detriment was observed in the ÖSSR condition, but it still did not provide a benefit to the higher mobility group in terms of laps completed or distance walked. While confounding factors such as the high intensity of the experimental protocol and the participants’ limited acclimation period with the Power Knee could have contributed to the lack of an endurance benefit, it is also possible that more inherent factors such as the added mass and cognitive load of interacting with the robotic prosthesis prevented users from performing more strongly in the Power Knee conditions.

The lower mobility group showed more mixed results, with some participants completing more laps with the Power Knee conditions. However, the variance was too high between conditions and participants to make any significant conclusions between conditions. Future studies with more lower mobility participants may be able to show clearer differences. Notably, despite the inconclusive quantitative results, two of the three lower-mobility participants procured their own Power Knee for everyday use following our study, suggesting a perception of substantial benefit.

### Clinical effect differences between Power Knee control policies

The two Power Knee control policies produced notable differences in both the data and in the participant feedback. Participants were, in general, able to complete more sit/stand cycles in the allotted time with the HKIC policy than with the ÖSSR policy (Fig. 4). The speed advantage of the HKIC policy compared to the ÖSSR policy during the actual standing motion was relatively small (Fig. 6), indicating that the larger speed benefits occurred during the sit/stand transitions and while sitting. In fact, the ÖSSR condition significantly slowed the sitting motions compared to both the HKIC and PRES conditions in the higher mobility group. This highlights one of the advantages of the continuously synchronized nature of the HKIC policy, as it allowed for faster transitions between sitting and standing by not requiring the users to perform discrete cues to trigger the behaviors. Some participants found the discrete classification cues for the ÖSSR policy difficult to master. Other sit/stand benefits were observed with HKIC, including the ability to switch from sitting to standing mid-way through the motion, which TF06 felt helped the prosthesis “feel like a real knee” (see video interview).

The HKIC condition also showed a gradual increase in the number of sit/stand cycles per set throughout the repeated sit/stand test (Fig. 9), which did not occur in either the ÖSSR or PRES conditions. This speed increase likely indicates that the participants were not fully acclimated to the HKIC behavior at the beginning of the experiment, and that they became more comfortable and effective over time. It is possible that the sit/stand benefits of the HKIC condition could have been stronger if the participants had been given more training than the single four-hour acclimation session. This hypothesis is supported by the participants’ qualitative feedback that indicated they felt they could perform even better with the HKIC condition after sufficient practice. This result highlights the importance of user acclimation, and suggests that longitudinal take-home studies may show clearer differences between conditions.

While walking, the participants generally felt that the HKIC condition was more dynamic and responsive compared to the ÖSSR condition, and that it provided them with more assistance. We believe that the sources of this sentiment are primarily the slightly higher early stance knee flexion (Fig. 13) and the reduced hip torque requirements, particularly for the higher mobility group (Fig. 18a, b). Interestingly, these perceived benefits did not manifest as notable changes in the selected walking speed (Fig. 16) or in the distance walked (Fig. 15). It is possible that other factors, including familiarity with walking on a treadmill, masked the differences between the two control policies and that different experimental protocols may be needed to elucidate their clinical effects.

The ÖSSR policy outperformed the HKIC policy in the incremental shuttle walk test by allowing nearly all participants to complete more laps than in the HKIC condition. The source of this difference is likely the speed at which the ÖSSR policy allowed users to transition from walking to sitting (Fig. 11). The HKIC policy required users to come to a complete stop before turning around and sitting, limiting their agility and top lap speed. Future work should investigate modifications to the HKIC mode selection algorithm [10, 24] in order to mitigate this limitation.

Similarly, participants found the ÖSSR controller’s walking behavior easier to learn and adapt to compared to the HKIC controller, with some participants commenting that it felt more similar to their passive microprocessor knees. Conversely, they reported that the HKIC condition required more time and practice to master, as its dynamic behavior differed significantly from what they were accustomed to with passive knees. Based on our observations during acclimation with the HKIC condition, we believe that this steeper learning curve could be due, in part, to the need for participants to unlearn compensatory habits that they had developed with passive knees. For example, participants who habitually applied strong hip flexion moments in early swing to overcome the lack of active knee control in their passive prostheses had a more difficult time learning how to walk smoothly with the Power Knee under HKIC control. TF03, who underwent amputation relatively recently, noted that recalling his pre-amputation walking gait helped him adapt to the HKIC condition, which felt to him like “like two legged walking” (see Additional file 2). Future work should consider how to lessen the HKIC controller’s learning curve, perhaps utilizing the training techniques presented in [44]. Additionally, future take-home studies that allow for much greater levels of acclimation, such as in [15], may reveal more distinct differences between control policies.

### The need for further clinical studies

The results of our laboratory-based experiments showed that a commercialized robotic knee prosthesis like the Power Knee can, under both traditional and novel control policies, produce significant clinical effects compared to the users’ prescribed passive knees. While some of these effects were negative (e.g., reduced agility and transition speed for higher mobility users, thermal limitations, etc.), many others were positive including improved standing time and symmetry during sit/stand for the lower mobility group, as well as increased toe clearance, increased ankle push-off moments, and reduced peak hip flexion moments while walking for the higher mobility group. However, it remains unclear whether or not these laboratory-based benefits would translate to longer-term health effects in real-life use cases. Further, it is unclear if the positive benefits would outweigh the negative effects we observed as well as other practical limitations of robotic prostheses such as audible noise. To answer these open questions, longitudinal studies utilizing robotic knees under various control policies should be performed, similar to [15]. The findings from these studies will be particularly informative for insurance coverage decisions; presently many insurers, including Medicare/Medicaid, only cover motorized knee prostheses for users with a K3 (mid/high) mobility level and a “documented comorbidity ...that impairs K3 level function with the use of a microprocessor-controlled knee alone” [3]. Future research could elucidate whether, for example, improvements in sit/stand symmetry via a robotic knee such as those observed in this study might allow a K3 user without an existing comorbidity to slow the onset of osteoarthritis. The results of these studies would provide practitioners and reimbursement agencies with important information about how and when robotic prostheses should be integrated into clinical practice.

### Study limitations

A primary limitation of this study was testing high-intensity activities over comparatively short durations, whereas real-world use cases are more likely to involve lower intensity activities over extended periods (e.g., full days at a factory job, walking between buildings on campuses, etc.). We made this experimental design concession in order to amplify any fatigue-related differences between conditions in order to allow their observation within a practical lab-based setting. While we believe that the fatigue effects caused by our experiments are a reasonable proxy for this more clinically-relevant use case, it is important to note that the high intensity adds confounding factors.

Specifically, the incremental shuttle walk test may have inadvertently tested agility more than fatigue, especially for the higher mobility group. As the cuing interval became very short, success was largely dependent on participants’ ability to transition quickly between walking and sitting. While this is a meaningful measure of the prosthesis’s usability, it does not directly quantify fatigue mitigation. Similarly, some participants struggled with fast walking on a treadmill for the full fifteen minutes, attributing it to poor medial-lateral balance, socket sweat/movement, and contralateral limb pain. These confounding factors may have masked the performance changes resulting from the different conditions. Additionally, the thermal limitations observed with the Power Knee in the HKIC condition for participant TF02 may have been due to the intensity of the experiments rather than a fundamental problem for his everyday use of the device. In typical activities of daily living, periods of high knee torque would likely be separated by longer breaks, allowing the knee sufficient time to cool.

Another limitation of this study is its relatively small sample size (n=7). Although we enrolled a diverse mix of participants, the division into two mobility groups resulted in small within-group sample sizes. The confidence intervals of some metrics were quite large, such as in the sit/stand symmetry metrics for the higher mobility group, and it is possible that higher numbers of participants could elucidate more subtle effects between conditions (especially between Power Knee controllers). Future work should enroll larger cohorts within each mobility level to increase statistical power. Nevertheless, some clear differences between the three conditions were still observed, despite the smaller sample sizes.

A related limitation is the considerable time and practice required to acclimate to different prostheses and control policies [45]. Although we provided a dedicated four-hour training session for each novel condition, full acclimation likely takes much longer. It is possible that better outcomes could be achieved in the two Power Knee conditions after more practice. Future take-home studies could address both of these limitations by allowing users ample time to practice with the different prostheses and control policies in more realistic settings, similar to [15].

Similarly, while our study compared two control approaches, robotic prosthesis control is a very active area of research with frequent innovations [46]. Our results showed significant relationships between the control policy used and the resulting clinical metrics. For example, the phase-based controller demonstrated greater reductions in hip torque compared to the ÖSSR controller, but the ÖSSR controller produced more rapid walk-to-sit transitions. This suggests future innovations around the polices tested in this work, as well as entirely different control approaches, may also offer unique benefits for different activities or user populations. Future clinical studies should evaluate new control approaches as they emerge to determine which control policies best serve specific user needs.

Finally, our qualitative participant feedback results should be interpreted cautiously, as participants can be biased towards enjoying new devices and can attempt to please the researcher by reporting what they think will be favorable opinions. While we attempted to elicit honest feedback by blinding the participant to the control policies used, it was impossible to blind the participants between the PRES and Power Knee conditions. Further, the researchers were not blinded when conducting the informal interviews, which did not follow a strict interview script. These factors could have introduced bias in the participant responses and in the subsequent analysis. Future studies with more systematic and formal qualitative assessments should be performed to validate our findings.

## Conclusions

In this study, we demonstrated that the Össur Power Knee can produce significant effects on users’ ability to perform sitting, standing, and walking activities in comparison to passive knees. The specific benefits we observed depended on both the user’s mobility level and the activity performed. Lower mobility users experienced improved inter-limb symmetry and faster standing speed during sit-to-stand transitions, while higher mobility users demonstrated increased toe clearance, more early-stance knee flexion, and reduced peak hip flexion moments during walking. Many participants perceived qualitative effort reductions with the Power Knee, though we found that the perceived and measured clinical benefits did not translate to increases in users’ ability to quickly perform repeated functional tasks or to walk at a fast pace for a long time. Other downsides of robotic knee use, such as reduced agility, slower transitions, and thermal limitations, were also observed with the Power Knee, primarily among the higher mobility users.

We also found that the phase-based and default FSM control policies each offered distinct trade-offs. The phase-based policy, while requiring more time to learn and sometimes producing slower walk-to-sit transitions, allowed the higher mobility group to complete more sit-stand cycles and often resulted in larger effect sizes in key clinical benefits like sitting knee moment symmetry, toe clearance, early-stance knee flexion, and peak hip moments. Conversely, the default control policy was easier to learn and often enabled more agile walk-to-sit transitions, but was generally more measured in its assistance with the higher mobility group. Some users preferred the default policy for its familiar behavior, but some also reported that, with time and practice, the phase-based policy delivered a greater sense of assistance and more natural movement. Future controller development should focus on balancing the strengths of each policy to maximize the Power Knee’s benefits for both mobility groups while minimizing drawbacks.

Overall, our findings motivate larger and longer-term studies to determine whether the effects observed in our lab-based experiments persist in real-world settings and what their long-term effects are on users’ overall health. Given that user outcomes were sensitive to both user mobility level and the control policy, future studies should explore both factors in order to develop a comprehensive understanding of the clinical effects of powered knees compared to passive MPKs. Ultimately, thorough investigations of the benefits and limitations of powered knee prostheses will be essential for informing clinical practice and supporting evidence-based decisions regarding insurance coverage and access.

## Supplementary Information

Additional file 1: Videos of selected experiments.

Additional file 2: Participant exit interviews.

## Data Availability

The datasets generated during and/or analyzed during the current study are available in the University of Michigan Deep Blue Data repository, https://doi.org/10.7302/yqhb-pj35.

## Appendix A

### Control policy details

#### A.1 Default control system

The Power Knee ships from the factory with a default control system that manages the motor’s behavior and enables the user to perform various activities of daily living. While the full details of the control system are proprietary and there is no specific public disclosure of how it operates, the published patents on the device [21, 22] give us an idea of how the controller is likely implemented. From our experience with the Power Knee, we believe that these patents reasonably describe the Power Knee’s default control logic.

At the lowest level, the motor’s torque command is calculated through feedback on the motor’s position, velocity, and the estimated joint torque:

A1 $$\begin{aligned} \tau = K_p(\theta _\text {d} - \theta ) + K_d(\dot{\theta }_\text {d} - \dot{\theta }) + K_a\hat{\tau }, \end{aligned}$$

where $K_p, K_d$, and $K_a$ are feedback gains and $\theta _\text {d}$ and $\dot{\theta }_\text {d}$ are position and velocity references, respectively. $\hat{\tau }$ is the estimated joint torque based on the measured deflection of the compliant transmission and its known elastic properties. By changing these gains and references, the control system can reproduce the different functions of the missing biological limb. For example, if $K_a = -1, K_p = 0, K_d = 0$, the system in theory becomes an infinite impedance, effectively locking the joint to support any applied load. Alternatively, if $K_a = 1$, the system should have zero impedance, allowing the natural free-swinging dynamics of the system to move the joint.

Based on various thresholds and triggers, the control software modifies the gains and references to produce the appropriate locomotion behaviors. For example, an FSM is used during walking that sequences through different control gain sets to render a high impedance early stance phase, an active flexion late stance phase, a low impedance early swing phase, and an energy absorbing late swing phase (Fig. 7 in [22]). These gains are likely scheduled with respect to various biomechanical measurements, such as walking cadence, to enable gait adaptation.

User-interpretable rules and cues dictate the transitions between ambulation modes (e.g., walking vs. sitting down), each with their own set of control gains. The user manual for the Power Knee describes the cues and motions required to trigger mode changes for various activities, such as standing from a chair [47]. A mobile-phone application allows a clinician to adjust various aspects of the controller, such as yielding resistance and standing assistance, and to initiate an automatic tuning procedure based on observations of a user’s gait.

#### A.2 Experimental phase-based control system (HKIC)

The HKIC controller uses continuous, data-driven models of joint impedance and kinematics to produce biomimetic joint behavior for various activities. It comprises two high-level modes: $\mu \in \{$Sit/Stand, Walk$\}$, which are automatically selected via a Finite State Machine (FSM). Simple, explainable transition rules based on the user’s thigh motion control the transitions between modes (see Fig. 1 in [10] for further detail). A time-based interpolation between modal torque commands is used to smooth transitions between control modes over 100 ms.

In addition to the discrete mode $\mu$, the controller calculates a continuous task variable $\chi$ and a continuous phase variable $\phi$ to evaluate the kinematic and impedance models (Fig. 19). In this initial translation to the Power Knee, we only include walking speed in the task variable $\chi$, though other variables such as ground incline could be included in future iterations [7]. The phase variable $\phi \in [0,1]$ quantifies the user’s progression through either a walking gait cycle [7] or sitting and standing motions [10]. We calculate $\phi$ based on algebraic mappings of the user’s thigh angle $\theta _\text {th}$, giving them indirect volitional control over the prosthesis’s behavior. The parameters used to calculate the phase variables adjust to the current task, allowing $\phi$ to appropriately parameterize the motion regardless of the varying thigh kinematics associated with different speeds and chair heights [28, 32].

While the user is in stance (which includes the entirety of Sit/Stand mode), the knee torque set point $\tau$ is calculated based on the joint angle $\theta$ and velocity $\dot{\theta }$ using impedance control:

A2 $$\begin{aligned} \tau = K(\phi , \chi , \mu ) \left[ \theta _\text {eq}(\phi , \chi , \mu ) - \theta \right] - B(\phi , \chi , \mu )\dot{\theta }, \end{aligned}$$

where the stiffness *K*, damping *B*, and equilibrium angle $\theta _\text {eq}$ are functions of the phase variable $\phi$, task variable $\chi$, and mode $\mu$. These parametric functions are fit offline to able-bodied training datasets [31, 32] such that (4) produces biomimetic joint torques when subjected to biomimetic joint kinematics. Details on the impedance model generation can be found in [7, 10].

Similarly, we use position control during the swing phase, where the desired joint angle $\theta _\text {d}$ and velocity $\dot{\theta }_\text {d}$ are modeled as functions of $\phi , \chi$, and $\mu$. This kinematic model is likewise generated based on able-bodied kinematic data via convex optimization, with details in [48]. The knee joint torque is then calculated through a proportional-derivative position controller as

A3 $$\begin{aligned} \tau = k_\text {p}[\theta _\text {d}(\phi , \chi , \mu ) - \theta ] + k_\text {d}[\dot{\theta }_\text {d}(\phi , \chi , \mu ) - \dot{\theta }] , \end{aligned}$$

where $k_\text {p}$ and $k_\text {d}$ are proportional and derivative gains, respectively. These gains are held constant across users.

In our previous work, we developed and evaluated our controllers using a prototype quasi-direct drive knee-ankle prosthesis [49]. The simple drivetrain dynamics of this prosthesis allow us to accurately control the joint impedance by assuming a proportional relationship between motor current and joint torque via the gear ratio and the motor’s torque constant. The Power Knee’s more complex drivetrain, however, requires us to consider its reflected inertia, friction, and series elasticity to achieve accurate impedance control. Additionally, it is well known in control theory that series elasticity can introduce stability problems when attempting to directly control joint position via non-collocated joint feedback [50]. Therefore, modifications to our original implementation are necessary in order to ensure accurate and stable control of the Power Knee.

We utilize the approach presented in [51] to calculate optimal motor feedback gains that account for the drivetrain dynamics and minimize the rendered impedance error. Instead of calculating a motor torque set point using (4) and (5) directly, we use a collocated motor-space controller of the form

A4 $$\begin{aligned} \tau = K_1({\theta }_\text {eq,m} +\theta ^* - \theta _\text {m}) - B_1\dot{\theta }_\text {m}, \end{aligned}$$

where $K_1$ and $B_1$ are feedback gains that depend on the desired impedance *K* and *B*, the system parameters (inertia, viscous damping, etc.), and the transmission stiffness at the current transmission deflection. The motor states $\theta _\text {m}$ and $\dot{\theta }_\text {m}$ are decoupled from the joint states $\theta$ and $\dot{\theta }$ via the transmission elasticity. An offset angle $\theta ^*$ is included to account for the transmission’s nonlinear torque-angle relationship. Details on the calculation of $K_1$, $B_1$ and $\theta ^*$ can be found in [51].

Another key difference between the Power Knee and our prototype prosthesis is that the latter had an actuated ankle joint, allowing both the knee and ankle to emulate normative biomechanics across a variety of activities [7, 10]. In contrast, the Pro-Flex LP foot used in conjunction with the Power Knee has fixed elastic properties that relate its ankle kinematics to ankle torques, thereby constraining the space of producible behaviors. In sit/stand in particular, normative able-bodied ankle mechanics deviate from this constraint curve, making the ankle necessarily behave non-biomimetically. This non-biomimetic behavior can lead to configurations where the application of knee extension torques is detrimental rather than beneficial, as discussed in [20]. For instance, applying large knee extension torques while the user is standing with a vertical shank can result in slipping and instability. To prevent applying knee extension torque in this unstable configuration, we scale the sit/stand stiffness and damping references with a unit logistic mapping of the user’s shank angle $\theta _\text {sh}$. The logistic mapping is defined as

A5 $$\begin{aligned} \xi (\theta _\text {sh}) = \frac{1}{1 + e^{1.5(\theta _\text {sh}+ 2.0)}}, \end{aligned}$$

where the angle constants have units of degrees. To prevent closed-loop oscillations that can occur from the interaction between the torque command and $\theta _\text {sh}$, we low-pass filter $\theta _\text {sh}$ prior to (7). Since users typically adjust their posture slowly before standing, a 1 Hz cutoff frequency is sufficient to filter the signal without causing noticeable response delay. As $\theta _\text {sh}$ increases above 0 (*i.e.* the ankle shifts anterior to the knee), the stiffness and damping asymptotically scale to 0, creating no knee joint torque. As $\theta _\text {sh}$ decreases beyond 0, the logistic function approaches 1, restoring the stiffness and damping commands to their normative values.

## Abbreviations

DoA: Degree of asymmetry
FSM: Finite state machine
GRF: Ground reaction force
HKIC: Hybrid kinematic impedance controller / HKIC condition
LMM: Linear mixed effects model
LP: Low profile
PRES: Prescribed condition
TUG: Timed up and go
ÖSSR: Össur condition
